# Pyometra Caused by an Adenoma Occluding the Cervix Uteri

**Published:** 1882-10

**Authors:** 


etc., which promise to be highly interesting. The first series begins with five lectures of the celebrated physiologist, F. M. Charcot. These lectures are truly scientific, without a particle of those mysterious sentences to be found in former lectures of Charcot. It seems that the French medical schools are reforming at the present time, and that they again aspire to the high position among medical schools which they occupied half a century ago. Prof. Charcot is doubtless on the path of true scientific research, as these lectures on the pathogenic conditions of albuminaria satisfactorily prove, and the editor of this series, Dr. Bourneville, who is also an earnest and active worker, is to be congratulated on these well selected publications. In our next number we will give some extracts from these lectures.

PYOMETRA CAUSED BY AN ADENOMA OCCLUDING THE CERVIX UTERI.

The patient, sixty-seven years of age, menstruated the last time fifteen years ago. For the last two years a bloody discharge has taken place from the vagina. The patient became reduced in strength. The uterus was movable, but the index finger could pass easily into the os externum. There was a fluctuating tumor, a part of which was extirpated instantly. The microscopical examination showed glandular elements, and proved the growth to be an adenoma of the mucous membrane of the cervix uteri. The patient not agreeing to a total extirpation, suffered for the next three months from severe pains in the suprapubic and right iliac regions, and the discharge had a fetid smell. A severe cough harrassed the patient continually. She at length resolved to undergo the operation. The body of the uterus was now much enlarged and somewhat elastic. The cervix was divided with scissors on both sides, the growth laid bare, and removed by scraping with the curette. When the upper part of the growth was removed, there was suddenly an escape of pus and blood to the amount of 100 ccm. The cavity was then scraped out, and a five per cent. carbolic solution injected. The walls contracted easily, and the woman was dismissed as cured after two weeks. The cough never reappeared from the day of the operation.—*Corresp. Blatt f. Schweizer Ærzte.*

